# A Circadian Trough in Glucocorticoid Signaling Is Essential for Bone Health in Mice

**DOI:** 10.1111/acel.70479

**Published:** 2026-04-09

**Authors:** Annelies E. Smit, Kaiming Yue, Sander Kooijman, Salwa Afkir, Maaike Schilperoort, Marijke Koedam, Bram C. J. van der Eerden, Jan Kroon, Onno C. Meijer, Elizabeth M. Winter

**Affiliations:** ^1^ Department of Medicine, Division of Endocrinology Leiden University Medical Center Leiden the Netherlands; ^2^ Leiden University Medical Center, Einthoven Laboratory of Experimental Vascular Medicine Leiden the Netherlands; ^3^ Department of Internal Medicine Erasmus Medical Center Rotterdam the Netherlands; ^4^ Department of Medicine, Center for Bone Quality Leiden University Medical Center Leiden the Netherlands

**Keywords:** aging, circadian rhythm, glucocorticoids, osteoporosis, translational research

## Abstract

We previously demonstrated that flattening circadian glucocorticoid (GC) rhythmicity without increasing overall GC exposure induces an osteoporotic phenotype in mice. Here, we aimed to further elucidate the importance of the amplitude and timing of circadian GC oscillations for bone health. C57Bl/6J mice were implanted with vehicle or corticosterone slow‐releasing pellets to flatten the circadian GC rhythm. To differentiate between the importance of circadian GC peaks or troughs, mice with flattened GC rhythm received daily CORT injections at the time of the natural GC peak, or glucocorticoid receptor (GR) antagonist (RU486) injections at the time of the trough. One week of flattened GC rhythm reduced serum bone formation marker P1NP levels. Reinstating a trough with RU486 rescued this loss, whereas reinstating a GC peak did not. In an experiment in which mice with flattened GC rhythm received prolonged treatment for 7 weeks with RU486 at Zeitgeber time (ZT) 1 versus 11, we found that P1NP levels peaked with RU486 regardless of time of injection, altogether suggesting that bone formation depends on transient withdrawals from GCs. Seven weeks of flattened CORT rhythm reduced total lean mass and induced cortical bone thinning and trabecular bone loss. RU486 injection at either timepoint prevented cortical bone decline. Notably, trabecular bone volume was only preserved when RU486 was injected at the time of the natural GC trough at ZT1. In conclusion, reinstating a trough in GR signaling at its natural time of the day suffices to prevent osteoporosis in mice under conditions of flattened GC rhythm.

## Graphical abstract

Flattening the circadian rhythm of endogenous glucocorticoids induces osteoporosis in mice. We demonstrate that reinstating a trough in glucocorticoid receptor signaling stimulates bone formation and suffices to prevent osteoporosis. Reinstating the trough at its natural time of day is needed for full protection.

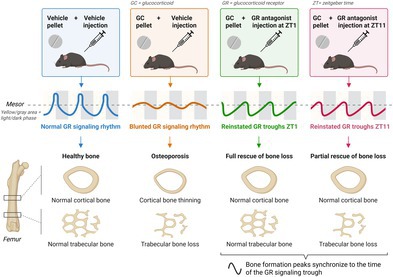

## 1 Introduction

Healthy bone tissue undergoes continuous remodeling, where the formation of new bone by osteoblasts is carefully balanced with the resorption of old bone by osteoclasts (Raggatt and Partridge 2010). When bone resorption exceeds formation, it results in a net loss of bone mass. This imbalance underlies osteoporosis, which is characterized by fragile bones that are prone to fractures due to low bone mass and poor structural integrity (van Staa et al. 2001). Osteoporosis risk increases with age. Globally, one in three women and one in five men over the age of 50 will experience an osteoporotic fracture in their remaining lifetime (Gonnelli et al. 2013; Kanis et al. 2000; Melton 3rd et al. 1992). Furthermore, the use of synthetic glucocorticoids (GCs) is a major risk factor for osteoporosis. Fractures are estimated to occur in 30%–50% of chronic GC therapy users, depending on age, and lead to pain, morbidity, and increased mortality (Angeli et al. 2006). With aging, circulating levels of cortisol the main endogenous GC in humans tend to rise, and GC therapy increases overall GC exposure. The adverse skeletal effects of GCs have traditionally been attributed to excessive GC levels, as fracture risk increases with GC dose (van Staa et al. 2001). However, the disruption of circadian GC rhythm with age (Veldhuis et al. 2013), and during GC therapy (Amin et al. 2014), may also contribute to the increased osteoporosis risk.

Like other metabolic processes, bone remodeling is tightly regulated in a circadian, that is, 24‐h, rhythm. Circulating levels of bone remodeling markers in humans (Redmond et al. 2016) and the expression of genes involved in bone remodeling in mice (Schilperoort et al. 2021) exhibit 24‐h oscillations. The intrinsic clock of bone tissue, based on core circadian regulatory or “clock” proteins, is critical to bone health, as global and osteoblast‐specific deletions of clock gene *Bmal1* lead to bone loss in mice (Samsa et al. 2016; Takarada et al. 2016). The peripheral clock in bone is synchronized to the central circadian clock located in the suprachiasmatic nucleus (SCN) of the hypothalamus (Welsh et al. 2010). As such, disruptions to the central circadian rhythm have been shown to impair bone remodeling in both mice and humans (Schilperoort et al. 2020; Feskanich et al. 2009; Quevedo and Zuniga 2010; Lucassen et al. 2016).

Peripheral clocks are synchronized to the central circadian rhythm through neuronal and hormonal signals, with endogenous GCs—predominantly cortisol in humans and corticosterone (CORT) in mice—playing a particularly important role. The SCN regulates GC secretion via the hypothalamus‐pituitary–adrenal (HPA) axis, resulting in rhythmic release of GCs from the adrenal gland (Czeisler 1995; Ishida et al. 2005). This rhythm is characterized by a peak in the hours before awaking (early morning for humans and evening for most rodent species), and a trough at the onset of the resting phase (Upton et al. 2023). Circulating GCs synchronize circadian rhythms in peripheral tissues by binding to the glucocorticoid receptor (GR) (Kalsbeek et al. 2011). In the absence of rhythmic GR activation, rhythmic expression patterns in bone are diminished (Fujihara et al. 2014). Disrupted rhythmic expression of circadian and bone function‐related genes was also observed in mice with flattened endogenous GC rhythm (Schilperoort et al. 2021). In this study, mice were implanted with slow‐release, low‐dose GC pellets, resulting in continuous GC exposure within the physiological range. Notably, the loss of circadian GC variation—without an increase in overall GC exposure—led to the development of osteoporosis, demonstrating the importance of GC rhythm to bone health.

Given the detrimental effects of a flattened GC rhythm on bone health, we aimed to further elucidate the mechanism by which circadian GC rhythm amplitude and timing regulate bone remodeling. Specifically, we investigated whether reinstating correctly timed oscillations in circulating GC levels can prevent an osteoporotic phenotype in mice.

## 2 Results

### 2.1 RU486 Antagonizes GR Signaling in Bone

**FIGURE 1:**
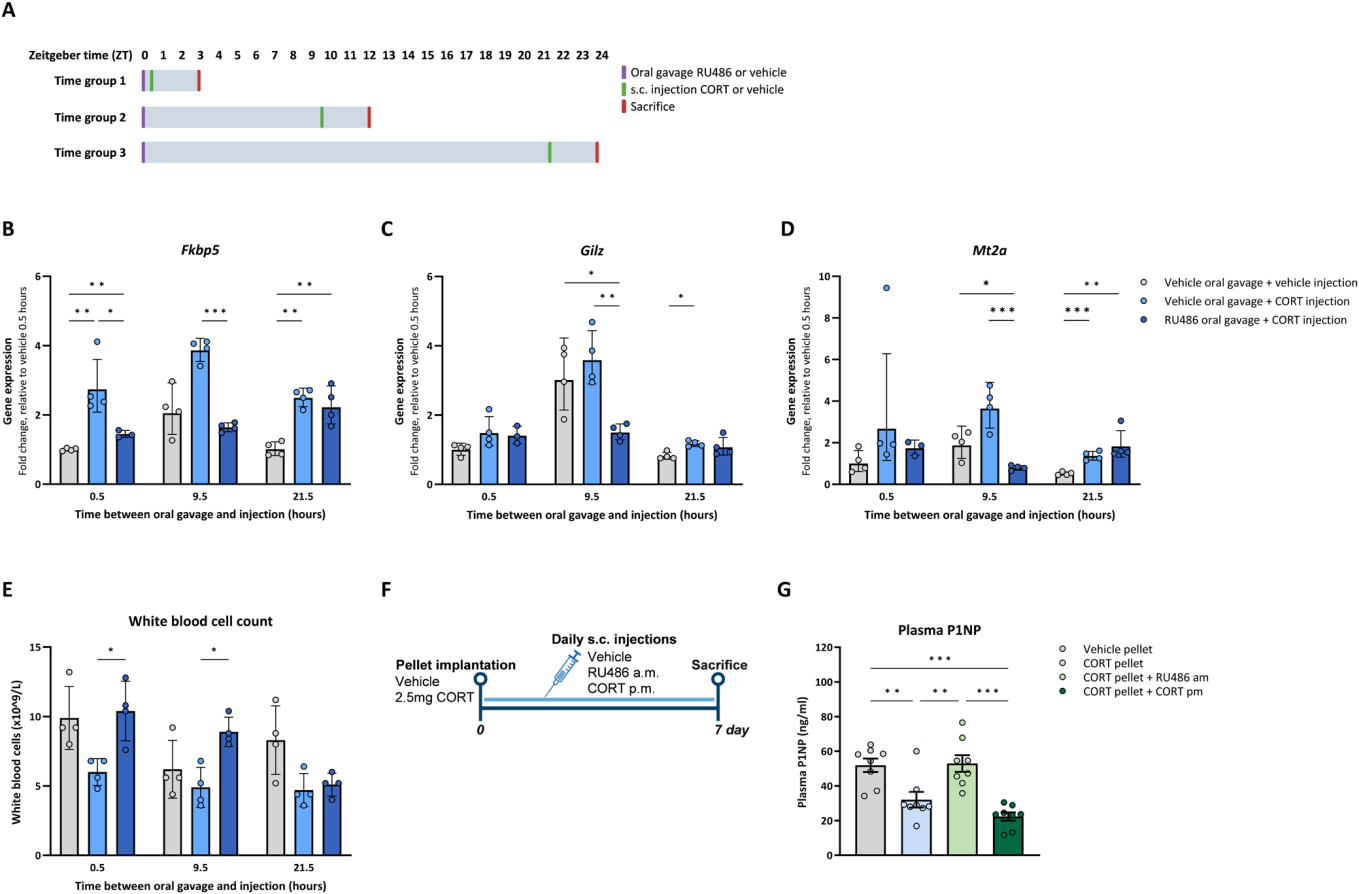
Reinstating a morning trough, but not an evening peak in GR signaling rescues bone formation in mice with flattened glucocorticoid rhythm. (A) Experimental set‐up to determine the duration of RU486‐mediated suppression of GR target genes *Gilz* (B), *Fkbp5* (C), *and Mt2a* (D), as well as its effect on white blood cell counts (E) in female C57BL/6 mice (*n* = 4 per treatment group). (F) Experimental set‐up to evaluate plasma bone formation marker P1NP levels (G) in CORT pellet‐implanted mice receiving daily injections with glucocorticoid receptor antagonist RU486 at the time of the natural GC trough (a.m.), or corticosterone (CORT) at the time of the natural GC peak (p.m.) in male C57BL/6 mice (*n* = 8/treatment group). Data represents means ± SEM, including individual data points. *p < 0.05, **p < 0.01, ***p < 0.001, according to one‐way ANOVA with Dunnet's post hoc test.

RU486 is a GR antagonist with a reported circulating half‐life of ~1.5 h in rodents (Deraedt et al. 1985). However, the functional duration of GR antagonism is not known. To determine how long RU486 antagonizes GR signaling in bone, we administered RU486 at zeitgeber time (ZT) 0, and CORT was injected 0.5, 9.5, or 21.5 h hereafter (Figure 1A). Expression of GR target genes *Fkbp5*, *Gilz*, and *Mt2a* showed diurnal variation in vehicle‐treated mice, coinciding with the endogenous glucocorticoid rhythm (Figure 1B–D), and CORT injections increased GR target gene expression. Pre‐treatment with RU486 for 0.5 h before CORT prevented induction of *Fkbp5*, while RU486 administered 9.5 h in advance antagonized the expression of *Fkbp5*, *Gilz*, and *Mt2a*. In contrast, RU486 given 21.5 h before CORT no longer suppressed the expression of any of the GR target genes. As an additional functional readout, we measured white blood cell counts, which also displayed a diurnal pattern in vehicle‐treated mice (Figure 1E). Consistent with the gene expression data, RU486 increased white blood cell counts when administered 0.5 or 9.5 h prior to CORT, but not when given 21.5 h in advance. In a separate experiment, we show that RU486 administered 0.5 h before CORT suppressed GR target gene expression in both cortical and trabecular bone (Figure S1A–D). Together, these data indicate that the functional effects of RU486 persist for at least 9.5 h and up to 21.5 h, suggesting that RU486 can be used to induce a trough in GR signaling.

### 2.2 Reinstating a Diurnal Trough in GR Signaling Rescues Bone Formation

We next evaluated whether bone formation responds to reinstated troughs or peaks in GC signaling rhythm (Figure 1F). Flattening of GC rhythm by CORT‐pellet implantation reduced plasma levels of the bone formation marker P1NP as compared to vehicle mice after 1 week (Figure 1G). This decrease in P1NP levels was prevented by daily injections with RU486 at ZT2, that is, around the time of the GR trough. In contrast, P1NP levels were not restored in mice receiving CORT injections at ZT7, that is, around the time of the GC peak. Together, these data indicate that the trough but not the peak of GC rhythm is important for bone formation.

### 2.3 Reinstating a Diurnal Trough in GC Signaling Rhythm Prevents an Osteoporotic Phenotype

**FIGURE 2:**
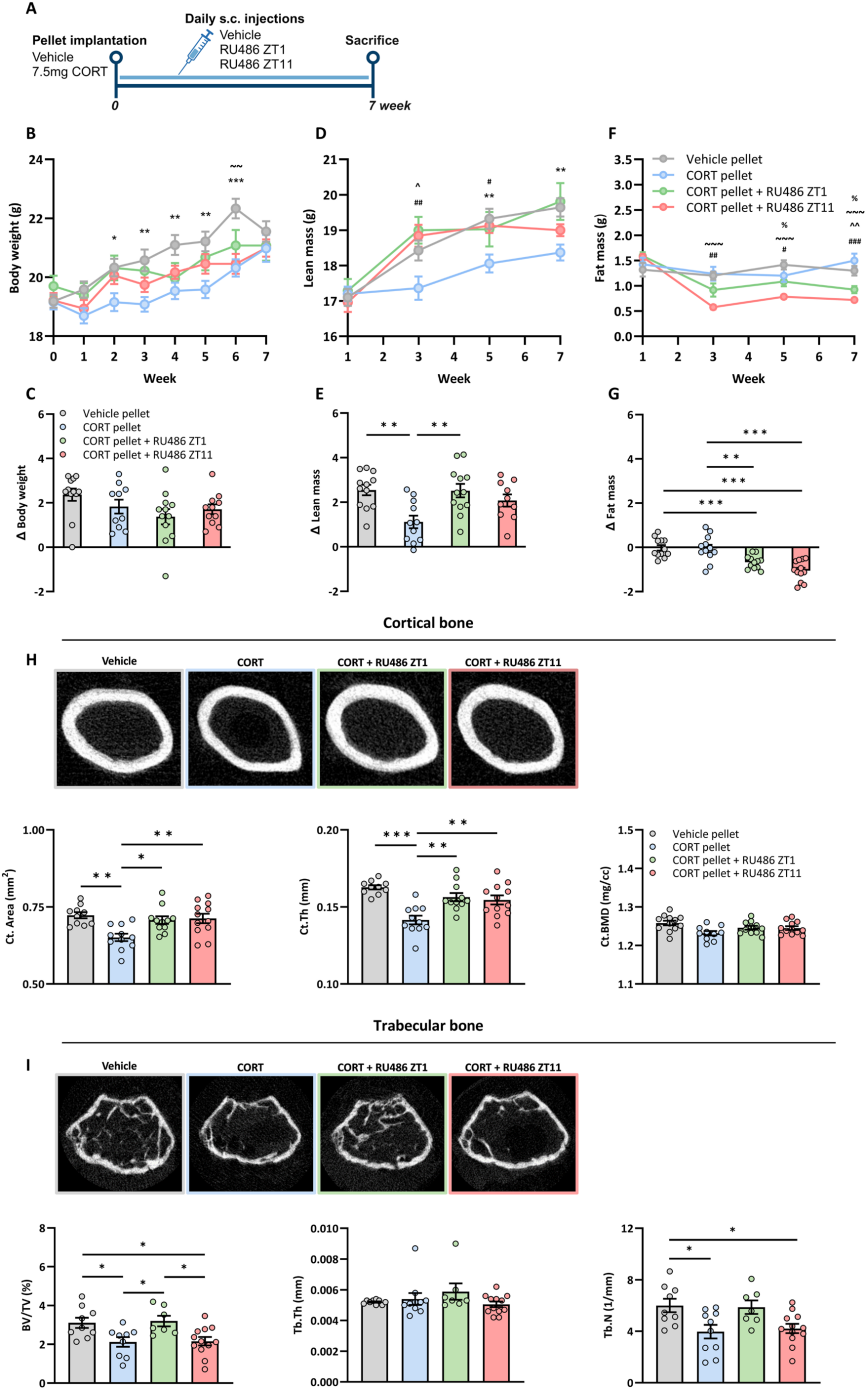
Reinstating a diurnal trough in GR signaling prevents an osteoporotic phenotype. (A) Experimental set‐up to determine long‐term effects on bone health of reinstating a diurnal trough in GR signaling at the time of the endogenous GC trough (ZT1) or peak (ZT11) in female C57BL/6 mice (*n* = 12/treatment group). Body weight (B), lean mass (D), and fat mass (F) by EchoMRI. (C, E, G) Delta values representing the difference in weights between endpoint and baseline. (H) Micro‐CT analysis and representative images of cortical bone to determine cortical bone area (Ct. Area), Cortical thickness (Ct.Th) and cortical bone mineral density (Ct.BMD). (I) Micro‐CT analysis and representative images of trabecular bone to determine trabecular bone volume fraction (BV/TV), trabecular thickness (Tb.Th) and trabecular number (Tb.N). Data represents means ± SEM, including individual data points. Pairwise comparisons panel B–D: * vehicle pellet versus CORT pellet, ~ vehicle pellet versus CORT pellet + RU486 ZT11, # CORT pellet versus CORT pellet + RU486 ZT11, % Vehicle pellet versus CORT pellet + RU486 ZT1, ^ CORT pellet versus CORT pellet + RU486 ZT1. *p < 0.05, **p < 0.01, ***p < 0.001, according to one or two‐way ANOVA with Dunnett's post hoc test.

We then investigated whether reinstating a trough in GR signaling rhythm prevents the development of an osteoporotic phenotype, as indicated by changes in body composition and bone microarchitecture (Figure 2A). We first validated the efficacy of our CORT pellets and confirmed that after 7 weeks, vehicle mice showed a pronounced circadian rhythm in circulating CORT, whereas mice implanted with CORT pellets exhibited a disrupted CORT rhythm characterized by a decreased amplitude and no significant difference between ZT2 and ZT12 (Figure S2A,B). RU486 treatment in CORT pellet‐implanted mice did not further influence CORT levels, and these remained significantly lower compared to vehicle mice at ZT12 (RU486 ZT11 −45%, *p* < 0.05; RU486 ZT1 −52%, *p* < 0.05).

CORT exposure reduced body weight from week 2 to 6, and RU486 administration at both ZT1 and ZT11 prevented this decline, resulting in comparable body weights across groups at week 7, as well as Δ body weights (Figure 2B,C). The reduced body weight upon CORT exposure was attributed to a reduced lean mass, also supported by a significantly lower Δ lean mass at endpoint (*p* < 0.01) (Figure 2D,E). RU486 at either time point prevented lean mass loss of CORT mice, and this resulted in a significantly higher Δ lean mass with RU486 at ZT1, with a similar trend for ZT11 (*p* < 0.06). Fat mass did not differ between vehicle and CORT mice, but RU486 at either time point decreased fat mass, which was more pronounced when given at ZT11 (Figure 2F,G). This effect occurred rapidly with a steep initial decline in fat mass until week 3, whereafter it remained mostly stable until week 7.

As expected, 7 weeks of a flattened CORT rhythm led to cortical thinning of the femur, as demonstrated by reduced cortical bone area and thickness as compared to vehicle mice (Figure 2H). RU486 treatment at either time point resulted in a similar cortical phenotype to vehicle mice, with significantly higher cortical bone area and thickness compared to vehicle‐injected CORT mice. Cortical bone mineral density did not differ across groups. Furthermore, CORT mice injected with vehicle had reduced trabecular bone volume and number, as compared to vehicle mice (Figure 2I). RU486 treatment at ZT1 prevented this decline, with trabecular bone volume and number comparable to vehicle mice, and significantly higher than vehicle‐injected CORT mice. In contrast, RU486 at ZT11 had no effect, as trabecular bone volume and number remained similar to vehicle‐injected CORT mice. The trabecular thickness did not differ across groups. Taken together, reinstating a diurnal trough in GC signaling rhythm with RU486 injection rescued the loss in lean mass and cortical bone irrespective of the time of the injection, whereas trabecular bone loss was only restored when the trough was introduced specifically at the timing of the natural GC trough.

### 2.4 Bone Remodeling Timing and Balance Depend on the Diurnal GC Signaling Trough

**FIGURE 3:**
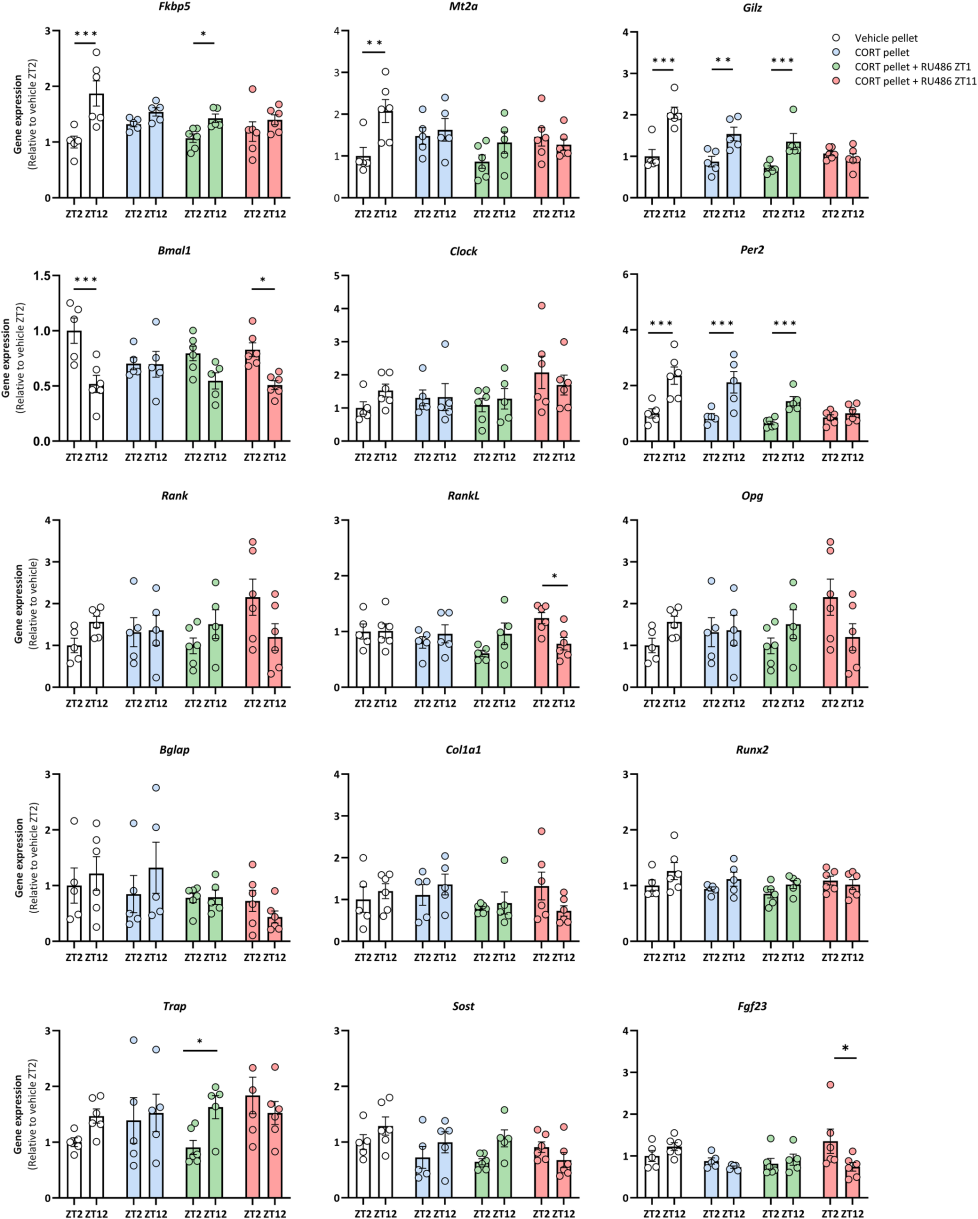
Reinstating a diurnal trough preserves the amplitude in GR response and clock gene expression, but does not alter expression of genes related to bone remodeling. Diurnal expression patterns of bone‐related genes (*Opg*, *Runx2*, *Bglap*, *Col1a1*, *cFos*, *Sost*, *Rank*, *Rankl*, *Trap*, *Ctsk*, and *Nfatc*), glucocorticoid‐response genes (*Fkbp5*, *Gilz*, *Sgk1*, and *Mt2a*) and clock genes (*Per1*, *Bmal1*, and *Clock*) in tibiae of vehicle or CORT pellet‐implanted female C57BL/6 mice, injected with vehicle, RU486 at ZT1 or RU486 at ZT11 for 7 weeks (*n* = 6/treatment group/timepoint). Data represent means ±SEM, including individual data points. *p < 0.05, **p < 0.01, ***p < 0.001, compared to vehicle or CORT group, according to one‐way ANOVA with Dunnett's post hoc test.

To investigate potential mechanisms underlying the detrimental effect of a flat CORT rhythm on bone microarchitecture, we analyzed expression profiles of genes involved in GR signaling, circadian regulation, and bone remodeling in tibiae collected at ZT2 and ZT12 (Figure 3). Vehicle mice showed a robust amplitude in canonical GR target genes *Fkbp5*, *Mt2A*, and *Gilz*. As expected, vehicle‐injected CORT mice had flattened diurnal variation in *Fkbp5* and *Mt2A* expression. RU486 treatment at ZT1, but not ZT11, restored the amplitude in *Fkbp5* expression. Conversely, *Gilz* expression rhythm was preserved in vehicle‐injected CORT mice but abolished by RU486 treatment at ZT11 but not ZT1. In addition, the diurnal variation in the circadian regulation gene *Bmal1* was flattened in vehicle‐injected CORT mice. RU486 treatment at either time point reinstated the diurnal amplitude, although statistical significance was only reached for the ZT11 group. The treatments did not affect the expression of the circadian gene *Clock*. Similar to *Gilz*, circadian gene *Per2* expression rhythm was maintained in vehicle‐injected CORT mice, and diminished by RU486 treatment at ZT11, but not ZT1. Genes involved in bone formation or resorption were not influenced by CORT pellets nor after RU486 injections at neither time of day. These data show that prolonged reinstatement of a diurnal trough in GR signaling in flattened GC rhythm reinstates the rhythm of several direct GR target and circadian regulation genes, while expression levels of genes that are more directly related to bone function were unaffected.

**FIGURE 4:**
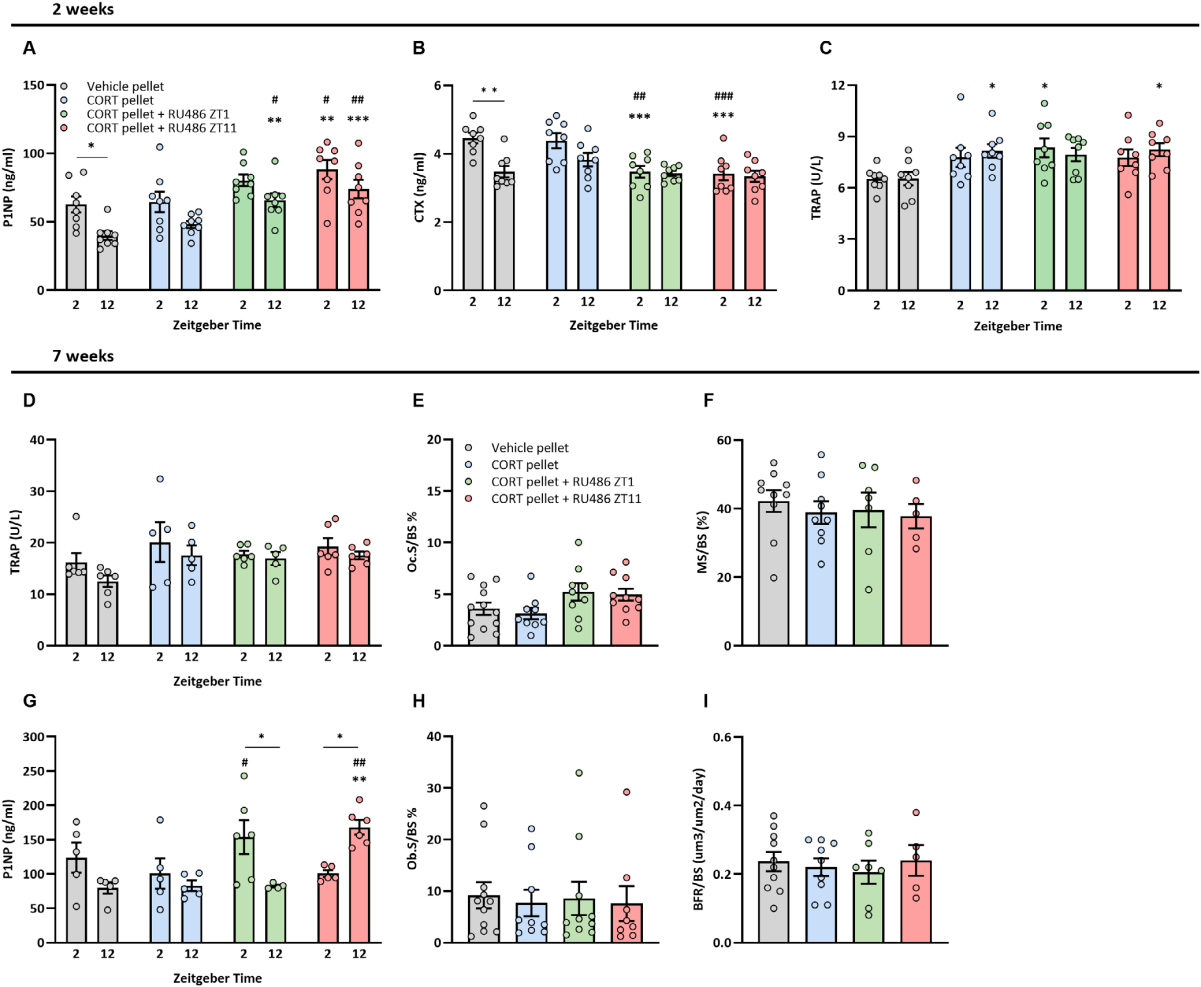
Bone remodeling responds to the trough in GR signaling rhythm. Plasma levels of procollagen type 1 amino‐terminal propeptide (P1NP; panel A, G), C‐telopeptides of type I collagen (CTX; panel B), and tartrate‐resistant acidic phosphatase (TRAP; panel C, D) at ZT2 and ZT12 in vehicle or CORT pellet‐implanted female C57BL/6 mice, injected with vehicle, RU486 at ZT1 or RU486 at ZT11 for 2 weeks in female C57BL/6 mice (*n* = 8/treatment group/timepoint) or 7 weeks (*n* = 6/group/timepoint). (E) Trabecular osteoclast surface area from femurs stained for TRAP and counterstained with Light Green. (H) Trabecular osteoblast surface area was determined from femurs stained for osteocalcin and counterstained with hematoxylin. (F, I) Calcein labeling determining mineralizing surface per bone surface (MS/BS) and bone formation rate per bone surface (BFR/BS). Data represents means ±SEM, including individual data points. Within‐group time point comparisons: *p < 0.05, treatment group comparisons: *p < 0.05, **p < 0.01, ***p < 0.001 compared to vehicle group, #*p* < 0.05, ##*p* < 0.01 compared to CORT group, according to one or two‐way ANOVA with Dunnett's post hoc test.

With regard to the levels and amplitude of circulating bone turnover markers, vehicle mice showed diurnal variation in plasma levels of osteoblast activity marker P1NP and osteoclast activity marker CTX. Plasma TRAP levels, which are indicative of osteoclast numbers, did not show a diurnal rhythm (Figure 4A–C). After 2 weeks, plasma TRAP levels of vehicle‐injected CORT mice were significantly increased compared to vehicle mice. Plasma CTX and P1NP levels remained unchanged upon CORT exposure, although the diurnal variation was reduced. RU486 treatment did not affect plasma TRAP levels, but did significantly reduce plasma CTX and increase plasma P1NP levels in comparison to vehicle‐injected CORT mice and vehicle mice. Together, these data suggest that reinstating a diurnal GR trough reduced osteoclast activity despite a higher osteoclast number, and increased osteoblast activity. After 7 weeks, plasma TRAP levels tended to appear higher in vehicle‐injected CORT mice as compared to vehicle mice, although not reaching significance (Figure 4D). No effect of CORT pellets or RU486 injections was observed on osteoclast surface area (Figure 4E). Against our expectations, vehicle‐injected CORT mice showed no significant changes in overall plasma P1NP levels (Figure 4G), or osteoblast surface area (Figure 4H). Notably, a peak in plasma P1NP levels was observed at ZT2 with RU486 treatment at ZT1, whereas P1NP levels peaked at ZT12 with RU486 treatment at ZT11, indicating that bone formation was synchronized to the timing of the trough in GC signaling. Finally, bone histomorphometry revealed no differences in bone formation rate across treatments (Figure 4F,I).

## 3 Discussion

In this study, we investigated the importance of the amplitude and timing of circadian oscillations in GC signaling to bone health. Flattening of the diurnal variation in CORT levels in this study led to cortical and trabecular bone loss, consistent with previous findings (Schilperoort et al. 2021). Reinstating a diurnal trough in GR signaling restored the balance in bone remodeling and prevented the deterioration of bone microarchitecture. The timing of the trough was crucial for full protection, as only treatment at the natural GC trough mitigated trabecular bone loss.

To re‐establish a trough in diurnal GR signaling, we administered GR antagonist RU486 to mice exposed continuously to low CORT levels via slow‐release pellets. RU486 suppressed GR response gene expression in bone between 9.5 and 21.5 h, resulting in a trough in GR signaling. In line, we observed reversed diurnal patterns of plasma bone formation marker levels and bone remodeling‐related gene expression after 7 weeks, corresponding to the timing of RU486 administration. Building on the premise of the ‘B‐flat’ model as first described by Akana et al. (1992) subtly elevated CORT levels during the natural trough trigger negative feedback on the HPA axis, resulting in the attenuation of the natural CORT peak. Importantly, the B‐flat model allows for the abolition of circadian variation in CORT levels without inducing CORT overexposure (Meijer et al. 2023). Consistent with previous studies using this model (Schilperoort et al. 2021; Kroon et al. 2021), the current study demonstrated that the model worked as expected, resulting in a flattened GC rhythm and no increase in the expression of GR‐target genes, indicating GC exposures within the physiological range.

Both insufficient and excessive GC exposure are detrimental to bone health by directly acting on the GR in bone cells. GR deficiency in osteoblast lineage cells causes loss of bone mass, while conversely it mitigates bone loss in mice treated with excess GCs (Rauch et al. 2010). Aside from insufficient or excessive GC levels, maintaining the circadian rhythm of GCs is crucial: flattening of the diurnal GC amplitude, even within normal GC ranges, has previously been shown to induce bone loss in mice (Schilperoort et al. 2021). Our study substantiates these findings, as disruption of the diurnal variation in CORT levels leads to loss of both cortical and trabecular bone mass. Given the systemic treatment, it remains unclear whether the flattened GC rhythm impacts bone directly or through other circadian or endocrine processes, such as changes in activity patterns or food anticipation.

Strikingly, while cortical bone loss was rescued by RU486 injection irrespective of timing, reinstating the diurnal GR trough prevented trabecular bone loss only when aligned with the natural GC trough. Similar differential effects between cortical and trabecular bone have been observed in other circadian rhythm interventions. For example, continuous light exposure reduces trabecular but not cortical bone mass in mice (). Furthermore, bone cells can respond differently to cues depending on their location: the beneficial effects of estrogen on bone are mediated via osteoblasts in cortical bone, while in trabecular bone this is mediated via osteoclasts (Manolagas et al. 2013). These findings suggest that the responsiveness of trabecular and cortical bone to rhythmic signals is regulated by distinct molecular pathways, which may also underlie their differing sensitivity to GR trough reinstatement. Future studies should directly compare how GR signaling rhythm regulates cortical versus trabecular bone remodeling through dynamic histomorphometry and gene expression analysis.

To explore mechanisms underlying these GC effects on bone parameters, we evaluated the balance in bone remodeling based on plasma markers and the expression of target genes. After 2 weeks, flattening of the corticosterone rhythm dampened the diurnal amplitude of the osteoblast activity marker P1NP and osteoclast activity marker CTX, while plasma TRAP levels were elevated indicating increased osteoclast numbers. These disturbances in bone remodeling are in accordance with our previous findings (Schilperoort et al. 2021). Reinstating a trough in GR signaling increased plasma P1NP and declined CTX levels, indicating a shift in the bone remodeling balance towards bone formation. However, plasma TRAP levels did not normalize towards values of vehicle mice. This may indicate involvement of the high‐affinity mineralocorticoid receptor (MR), which is activated by CORT but not antagonized by RU486. A role of the MR in GC‐induced osteoporosis has indeed been proposed before (Fumoto et al. 2014). It would therefore be worthwhile to investigate whether reinstating a trough in MR signaling would reduce osteoclast numbers.

After prolonged (i.e., 7 weeks) reinstatement of the trough in GC signaling, the peak in the bone formation marker P1NP synchronized with the time of RU486 injection, that is, the timing of the GC trough. While the overall level of gene expression was not affected, a similar opposite rhythmic expression pattern was found for several genes involved in bone remodeling. Furthermore, the rhythmic expression of circadian regulation gene *Bmal1* was reinstated, regardless of timing of the trough. Maintaining (rhythmic) expression of this gene may be of particular importance to bone, as global as well as osteoblast‐specific *Bmal1* deficiency induces bone loss (Samsa et al. 2016; Zhou et al. 2018). The fact that we observed changes in the rhythm of bone remodeling plasma and gene expression markers, but not in their overall levels after 7 weeks, may indicate that not only total bone remodeling activity but also the rhythmic patterns in which they occur are relevant to bone health. However, findings are complicated by potential differences in mRNA stability, variation in phase of expression patterns between genes (Schilperoort et al. 2021), and timing of the RU486 treatment relative to the time of tissue collection. More refined techniques, such as selective GR blockade in specific bone cell types or single‐cell RNA sequencing, could provide deeper insights into the distinct roles on osteoblasts, osteoclasts, and other bone cell populations in GR rhythm‐regulated bone remodeling. Nonetheless, these results demonstrate that bone remodeling is under control of the trough in GR signaling.

Similarly to our study design, the circadian variation in GC levels is flattened with advanced age and during synthetic GC therapy. During aging, the rhythmic activity of the suprachiasmatic nucleus (SCN) weakens and becomes less responsive to external cues, leading to a dampened cortisol diurnal amplitude (Veldhuis et al. 2013; Nakamura et al. 2016). Moreover, most synthetic GCs have a long biological half‐life, which suppresses the rhythmic release of endogenous GCs throughout treatment (Amin et al. 2014; Charmandari et al. 2014). It was previously thought that the harmful effects of GCs on bone are due only to an excess in GC exposure. Indeed, a dose‐dependent effect is well‐documented in both animal and population studies, but nonetheless even low doses of prednisolone can already elevate fracture risk (Van Staa et al. 2000). Our results indicate that administering a GR antagonist at the onset of the resting phase to mimic a trough in GC signaling may be a viable strategy to prevent bone loss. In the context of GC therapy, brief diurnal interruptions may allow sufficient GC exposure to preserve desired anti‐inflammatory effects of GC therapy while maintaining diurnal GC signaling. For aged individuals in whom the endogenous GC release is disrupted, an alternative approach could involve short, timed inhibition of cortisol synthesis. This could be achieved with, for example, 11b‐hydroxylase inhibitors, which have been shown to restore normal cortisol rhythm in patients with adrenal incidentalomas (Debono et al. 2017).

Our study has some limitations. First, although the CORT amplitude was not fully abolished by the CORT pellets, morning and evening plasma CORT levels were comparable. Pellets were replaced every 2 weeks throughout the study to ensure proper functioning, with the last replacement occurring 7 days before endpoint when plasma was drawn for CORT analysis. This may result in an underestimation of the effect of a reduced diurnal CORT amplitude on transient outcomes as plasma bone remodeling markers, dynamic histomorphometry, and gene expression patterns. Importantly, the validity of the model is substantiated by an earlier publication of our group, in which we demonstrated a decline in bone formation and increase in bone resorption with a fully flattened corticosterone rhythm after 7 weeks of equally‐dosed corticosterone pellet implantation (Schilperoort et al. 2021). We did not observe robust changes in bone remodeling at 7 weeks, which could be explained by recovery of bone tissue. Notably, we observed the largest difference in body weight and lean mass between CORT and vehicle mice early on in the study, and these differences were less pronounced at endpoint. In line, the CORT pellet‐induced reduction in trabecular bone volume was driven by a decrease in trabecular number while trabecular thickness was similar across groups. As lost trabeculae are typically not restored while remaining trabeculae can rethicken, the trabecular bone phenotype is in line with the unchanged trabecular bone formation rate. One difference between our current and previously published 7‐week flattened CORT rhythm experiment is the age of mice at baseline (respectively 7 weeks and 12 weeks). While unknown, mice may respond differently to prolonged disturbance of flattened glucocorticoid circadian rhythm depending on their age (Schilperoort et al. 2021). Furthermore, the evaluation at only two time points at endpoint may not have fully captured effects of our treatments on rhythmicity of mRNA markers. Additional timepoints may have been necessary to capture the peaks and troughs in gene expression patterns. Moreover, the group size of mice receiving RU486 injections at ZT1 was smaller than the other treatment groups, due to the exclusion of bones with incomplete epiphyses, which arose from inaccuracies during the bone isolation process. Nonetheless, the increase in trabecular bone volume and number compared to vehicle‐treated CORT mice was statistically significant, indicating that the effect size was large enough to retain sufficient statistical power. Finally, we focused on female mice, since females are more susceptible to osteoporosis than males (Alswat 2017). Nonetheless, we could rescue acute disturbances in bone remodeling in both male (Figure 1G) and female mice (Figure 4B–D). Repeating the current study in males would be worthwhile to elucidate potential sex‐specific differences.

In conclusion, we demonstrated that preserving a diurnal trough in GR signaling effectively prevents the loss of bone mass and structural integrity associated with a flattened GC rhythm. These findings not only enhance our understanding of how the rhythm in GR signaling regulates bone health but also suggest a novel therapeutic approach to combat osteoporosis in circumstances where GC rhythm is disrupted, such as during synthetic GC therapy and aging.

## 4 Experimental Procedures

### 4.1 Animal Experiments

Experiments have received approval from the Central Animal Experiments Committee in The Netherlands. C57BL/6 mice (Charles River Laboratories) were group housed under a standard 12 h:12 h light:dark regimen. Mice were fed standard chow diet *ad libitum*.

### 4.2 Corticosterone Pellets

Slow‐release pellets (1 cm in diameter and 4 mm thick) were made by compressing 2.5 mg (male mice) or 7.5 mg (female mice) CORT (27840, Sigma‐Aldrich) with cholesterol (100 mg in total) by using a TDP 0 Desktop Tablet Press (LFA Machines Oxford). Vehicle pellets consisted of 100 mg cholesterol. The constant release of CORT from the pellets suppresses the endogenous diurnal secretion of endogenous GCs, resulting in constant, arhythmic, circulating CORT levels without increasing total CORT exposure (Schilperoort et al. 2021; Akana et al. 1992; Kroon et al. 2021).

### 4.3 Animal Procedures

Mice were injected subcutaneously with buprenorphine (Temgesic, RB Pharmaceuticals Ltd) for analgesia and anesthetized using isoflurane inhalation. Pellets were implanted subcutaneously in the neck after making a small incision in the skin. In the longer experiment, pellets were replaced every 2 weeks to prevent encapsulation that would result in reduced corticosterone release. Every week, total body weight, lean mass, and fat mass were measured with an EchoMRI‐100 body composition analyzer (Echo‐MRI). Blood for plasma CORT measurements was collected into capillaries by making a small incision in the tail vein within 2 min after picking up the animal, to ensure reliable CORT levels before stress‐induced rises (Spiga et al. 2014). For fluorescent double labeling of bone, mice were injected intraperitoneally with 15 mg/kg calcein (C0875, Sigma‐Aldrich) in 0.2% sodium bicarbonate 8 and 2 days prior to sacrifice. At the end of the experiments, mice were killed through CO_2_ inhalation, blood was collected through heart puncture, and mice were perfused for 5 min with ice‐cold phosphate‐buffered saline (PBS). Then, tissues were isolated for further analysis.

### 4.4 Duration of GR Antagonism by RU486

To examine the duration of GR antagonism by RU486, eight‐week‐old female C57Bl/6J mice were randomized to receive either 30 mg/kg RU486 or vehicle through oral gavage. Subsequently, mice received a subcutaneous 3 mg/kg CORT injection or vehicle injection as control at 0.5, 9.5, or 21.5 h after RU486 administration (*n* = 4/treatment group). All mice were sacrificed 2.5 h after the subcutaneous injection.

### 4.5 RU486 Efficacy in Cortical and Trabecular Bone

To examine whether RU486 has efficacy as a GR antagonist in both cortical and trabecular bone, 9‐week‐old male C57Bl/6J mice were randomized to receive either 60 mg/kg RU486 or vehicle through oral gavage. 0.5 h after RU486 administration, male mice received a subcutaneous 3 mg/kg CORT injection or vehicle injection as control (*n* = 4/treatment group). All mice were sacrificed 2.5 h after the subcutaneous injection.

### 4.6 Peak and Trough Reinstalment

To evaluate whether bone formation responds to peaks or troughs in GR signaling, nine‐week‐old male C57Bl/6J mice were subcutaneously implanted with either vehicle or 2.5 mg CORT slow‐release pellets to flatten diurnal variation in CORT levels (*n* = 8/treatment group). To reintroduce a trough in GR signaling, mice received a subcutaneous injection of 30 mg/kg RU486 at ZT2 (i.e., around the time of the endogenous GC trough) and to reintroduce a peak mice received a subcutaneous 1.5 mg/kg CORT injection at ZT7 (i.e., around the time of the endogenous GC peak). Control groups, implanted with either vehicle or CORT pellets, received vehicle injections at ZT7. After 7 days, mice were sacrificed at ZT7.

### 4.7 A Timed Trough and Osteoporosis

To examine whether reinstating a diurnal trough in GR signaling prevents osteoporosis development and whether timing of the trough is relevant, 7‐week‐old female C57Bl/6J mice were implanted with vehicle or 7.5 mg CORT slow‐release pellets. Mice received daily subcutaneous injections with 30 mg/kg RU486 or vehicle. To evaluate the importance of timing, RU486 was injected either at the time of the endogenous GC trough (ZT1) or at the time of the endogenous GC peak (ZT11). After 7 weeks, mice were sacrificed at either ZT2 or ZT12 (*n* = 7/treatment group/time point). Subsequently, tibiae were isolated for gene expression analysis, femora were isolated for micro‐CT, calcein double labeling, and histological analysis.

To evaluate the short‐term effects of a reinstated diurnal trough in GR signaling on bone remodeling, 12‐week‐old female C57Bl/6J mice were implanted with vehicle or 7.5 mg CORT slow‐release pellets (*n* = 8/treatment group/time point). Mice received daily subcutaneous injections with vehicle or 30 mg/kg RU486 at either ZT1 or ZT11. After 2 weeks, mice were fasted for 4 h, and subsequently sacrificed at ZT2 or ZT12 (*n* = 7/treatment group/time point).

### 4.8 Bone Microarchitecture

To evaluate changes in bone microarchitecture, femurs were scanned at a resolution of 9 μm, using a SkyScan 1076 system (Bruker, Kontich, Belgium). According to the published guidelines (Bouxsein et al. 2010), the following settings were used: x‐ray power and tube current were 40 kV and 250 μA, respectively. Beam hardening was reduced using a 1‐mm aluminum filter, exposure time was 2.3 s, and an average of three pictures was taken at each angle with a rotation step angle of 0.8°. Segmentation of the reconstructed images was done on basis of global thresholding. Using software packages from Bruker (NRecon, CtAn, and Dataviewer), trabecular and cortical bone parameters were assessed. For femoral trabecular bone, a total of 50 slides (0.01 mm per slide) starting 100 slides above the growth plate were analyzed. For femoral cortical bone, 15 slides above and 15 slides below the midpoint of bone were analyzed. Using the same dataset, cortical bone mineral density was evaluated, using two reference phantoms with known hydroxyapatite concentration. Trabecular volume of interest was drawn manually, cortical volume of interest was determined automatically by analysis software.

### 4.9 Plasma Biochemistry

Blood collected in EDTA‐coated capillaries was centrifuged for 10 min at 4°C. The plasma top layer was transferred to Eppendorf tubes and stored at −80°C. Plasma CORT levels were measured using an ELISA kit, according to the manufacturer's protocol (corticosterone EIA, Immunodiagnostics). Plasma concentrations of bone resorption marker tartrate‐resistant acid phosphatase (TRAP) and bone formation marker procollagen type I N‐propeptide (P1NP) were determined using enzyme immunoassay kits (IDS) (Immunodiagnostics). Plasma concentrations of bone resorption marker carboxy‐terminal collagen crosslinks (CTX) were determined using an enzyme‐linked immunosorbent assay (ELISA) (Thermo Fischer). All assays were performed according to the manufacturer's instructions.

### 4.10 White Blood Cell Count

Directly following blood collection during sacrifice, 25 μL of whole blood was diluted in 75 μL PBS and kept on ice for 15 min. Hereafter, white blood cell counts were determined using the Sysmex XT‐2000iV system.

### 4.11 Histology

Femurs were fixed in 4% phosphate buffered formalin and transferred after 24 h to 10% EDTA for decalcification and stored at 4°C. After 3 weeks, bones were embedded into paraffin and cut into 5 μm sections. Sections were stained for TRAP using an acid phosphatase kit (Sigma‐Aldrich) and counterstained with Light Green. Osteoblasts were visualized by staining with primary anti‐osteocalcin antibody (ALX‐210‐333, 1:1000; Enzo Life Sciences), combined with secondary goat anti‐rabbit antibody (Dako), counterstained with hematoxylin. Sections were imaged using Philips Digital Pathology Solutions (PHILIPS Electronics). Osteoclast and osteoblast surface areas were quantified using TrapHisto software (van't Hof et al., 2017).

### 4.12 Calcein Labeling for Dynamic Bone Remodeling

Femurs were fixed in 4% phosphate buffered formalin. After 24 h, they were transferred to 70% EtOH and stored at 4°C. Subsequently, femurs were embedded into methyl methacrylate (MMA) and cut into 7 μm sections. Slices were counterstained using calcein blue (M1255, Sigma‐Aldrich), and imaged using a LEICA DMI6000 (Leica, Microsystems). In the trabecular bone area (starting 50 nm from the growth plate), the mineralizing surface per bone surface (MS/BS) and bone formation rate (BFR/BS) were determined using CalceinHisto software (van't Hof et al., 2017).

### 4.13 Gene Expression Analysis

Both ends of tibiae were cut off and bones were flushed with PBS to remove bone marrow before storage in RNAlater (Thermo Fisher) at −20°C. Bone tissue was homogenized mechanically (FastPrep‐24 5G bead beating grinder) in TRIzol RNA isolation reagent (Thermo Fisher) and stainless‐steel beads (Qiagen). Subsequently, RNA was isolated according to the manufacturer's protocol. Concentration of isolated RNA was measured by NanoDrop (Thermo Fisher). 100–500 ng RNA was reverse transcribed to cDNA with M‐MLV Reverse Transcriptase (Promega). qRT‐PCR was performed using a SYBR Green mix (Promega) on a CFX96 PCR machine (Bio‐Rad) to determine expression levels of GR response, clock, and bone‐related genes. Expression levels were normalized against the housekeeping gene *β*‐*Actin*.

### 4.14 Statistical Analysis

Statistical analyses were performed using Graphpad Prism 11.01 Graphpad Software Inc., San Diego, CA, USA. Significance was evaluated by either one‐way or two‐way ANOVA followed by Dunnett's or Bonferroni post hoc testing. A *p* value of < 0.05 was considered statistically significant.

## Author Contributions

A.E.S. performed experiments, analyzed data, and drafted the manuscript together with O.C.M. and E.M.W. K.Y., S.K., S.A., and J.K. performed experiments. M.K. and B.E. performed plastic tissue embedding and micro‐CT scans and guided subsequent data analysis. S.K., J.K., O.C.M., and E.M.W. helped to conceptualize the project and supervised the project. All authors critically reviewed the manuscript.

## Funding

This work was supported by Leiden University Fund (W20805‐4‐SPL); ZonMw (09150172310007).

## Conflicts of Interest

The authors declare no conflicts of interest.

## Supporting information


**Figure S1:** RU486 antagonizes the glucocorticoid receptor in cortical and trabecular bone. (A) Experimental set‐up to measure cortical and trabecular expression of glucocorticoid receptor‐response genes *Gilz* (B), *Fkbp5* (C), and *Mt2a* (D) in male C57BL/6 mice (*n* = 4/treatment group). Data represents means ± SEM, including individual data points. *p < 0.05, **p < 0.01, ***p < 0.001, according to one‐way ANOVA with Dunnet's post hoc test.

**Figure S2:** Slow‐release corticosterone pellets flatten the diurnal variation in plasma corticosterone levels. (A) Plasma levels of Corticosterone (CORT) at ZT2 and ZT12 in vehicle or CORT pellet‐implanted female C57BL/6 mice, injected with vehicle, RU486 at ZT1 or RU486 at ZT11 for 7 weeks. (B) Delta values representing the difference in plasma CORT levels at ZT2 and ZT12 (*n* = 6/group/timepoint). Data represents means ± SEM, including individual data points. **p < 0.01, ***p < 0.001, according to one‐way ANOVA with Dunnet's post hoc test.

## Data Availability

All data reported in this paper will be shared upon request, by the lead contact Elizabeth M. Winter (e.m.winter@lumc.nl). This paper does not report original code. Any additional information required to reanalyse the data reported in this paper is available from the lead contact upon request.
