# Supplementary figures and images for: A Circadian Trough in Glucocorticoid Signaling Is Essential for Bone Health in Mice
Source: Aging Cell. 2026 Apr 9;25(4):e70479. doi: 10.1111/acel.70479 (PMC13062760; doi:10.1111/acel.70479)

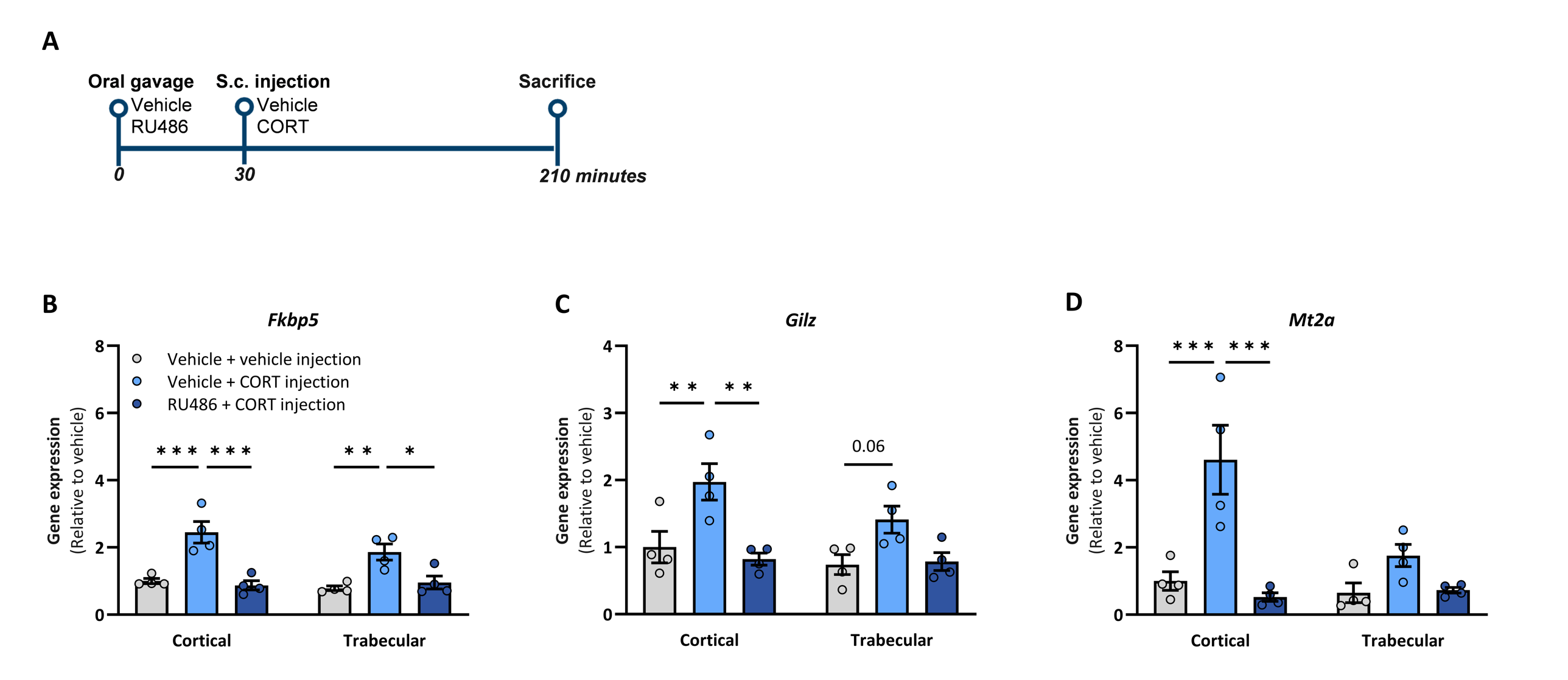

Supplement: Supplementary file 1 — Figure S1: RU486 antagonizes the glucocorticoid receptor in cortical and trabecular bone. (A) Experimental set‐up to measure cortical and trabecular expression of glucocorticoid receptor‐response genes Gilz (B), Fkbp5 (C), and Mt2a (D) in male C57BL/6 mice (n = 4/treatment group). Data represents means ± SEM, including individual data points. *p < 0.05, **p < 0.01, ***p < 0.001, according to one‐way ANOVA with Dunnet's post hoc test. [file ACEL-25-e70479-s001.tif]

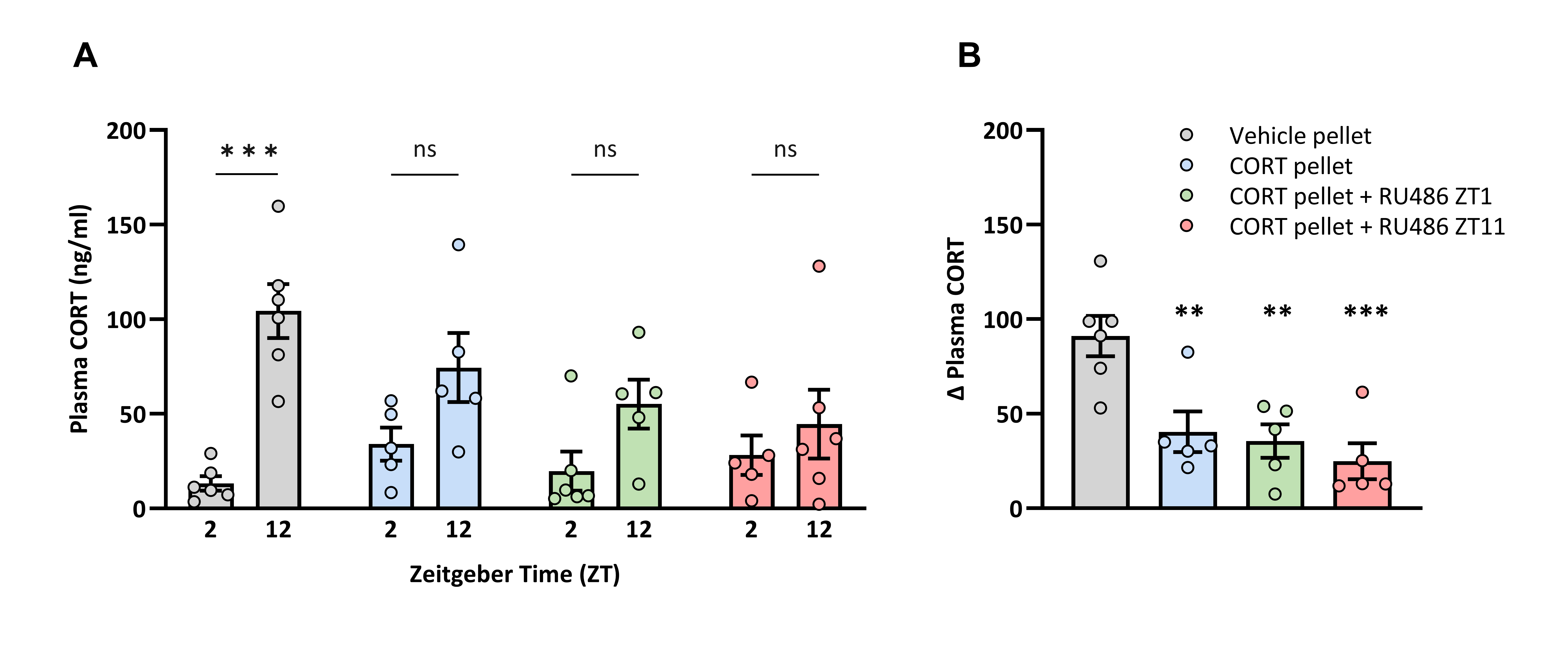

Supplement: Supplementary file 2 — Figure S2: Slow‐release corticosterone pellets flatten the diurnal variation in plasma corticosterone levels. (A) Plasma levels of Corticosterone (CORT) at ZT2 and ZT12 in vehicle or CORT pellet‐implanted female C57BL/6 mice, injected with vehicle, RU486 at ZT1 or RU486 at ZT11 for 7 weeks. (B) Delta values representing the difference in plasma CORT levels at ZT2 and ZT12 (n = 6/group/timepoint). Data represents means ± SEM, including individual data points. **p < 0.01, ***p < 0.001, according to one‐way ANOVA with Dunnet's post hoc test. [file ACEL-25-e70479-s002.tif]
